# Effect of a Trace Mineral Injection on Performance and Trace Mineral Status of Beef Cows and Calves

**DOI:** 10.3390/ani11082331

**Published:** 2021-08-07

**Authors:** Carmen J. Willmore, John B. Hall, Mary E. Drewnoski

**Affiliations:** 1 Department of Animal and Veterinary Science, University of Idaho, Moscow, ID 83844, USA; cwillmore@uidaho.edu (C.J.W.); jbhall@uidaho.edu (J.B.H.); 2 Department of Animal Science, University of Nebraska, Lincoln, NE 68583, USA

**Keywords:** cattle, conception, growth, liver, trace mineral

## Abstract

**Simple Summary:**

Often the forage alone does not provide enough trace minerals to meet the needs of grazing cows and their calves, specifically copper and zinc are almost always deficient, with Se varying among geographical locations. The trace mineral status of cows can affect their ability to get and stay pregnant as well as the health of their calf in early life. Injectable trace minerals can provide additional supplemental trace minerals and allow for a known amount of trace minerals to be delivered to each animal. The objective of this trial was to determine the effects of a trace mineral injection on the trace mineral status of cows and calves and the resulting impacts on the reproductive performance of the cows and the growth of their nursing calves. In this study, use of an injectable trace mineral increased the cow’s trace mineral stores, but the non-injected cows had adequate copper and zinc status due to free-choice mineral consumption, and no performance benefits of an injectable trace mineral were observed.

**Abstract:**

The objective was to determine the effects of an injectable trace mineral (TMI; Multimin 90) containing copper (Cu), manganese (Mn), selenium (Se), and zinc (Zn) on trace mineral status and the resulting impacts on reproduction of beef cows and the growth of their calves. Beef cows (n = 200) were assigned to receive TMI or no injection (CON) prior to calving and breeding over two consecutive years. Calves born to cows receiving TMI also received TMI at birth in both years and at 49 ± 1.3 days of age in year 1. The TMI increased (*p* = 0.01) liver Zn and tended (*p* = 0.06) to increase liver Cu concentrations. Short-lived effects of TMI on Se were observed. Liver Cu and Zn would have been considered adequate and Se marginal in the CON. Pregnancy due to artificial insemination and overall pregnancy rate did not differ (*p* ≥ 0.36) between treatments. Use of TMI did not increase calf pre-weaning gain. These data indicate that TMI does not improve the reproductive performance of beef cows with adequate trace mineral status or the pre-weaning performance of their calves.

## 1. Introduction

Forage is the primary component of beef cows’ diets; however, forage often does not provide adequate amounts of trace minerals; therefore, mineral supplementation is often required [1]. Copper and zinc are widely deficient in forages across the U.S., and the selenium content of forages is quite variable with some locations having extreme deficiencies and others being sufficient [2]. The trace mineral status of cows at calving can affect the calf’s mineral status at birth and its health in early life [3]. Additionally, a cow’s reproductive performance can be impacted by her trace mineral status [4]. Specifically, the trace minerals Cu, Se, and Zn play important roles in reproduction [3].

The most common form of mineral supplementation in grazing systems is free-choice mineral [5]. Typically, a cow’s appetite for a free-choice mineral is not related to her mineral requirement, but rather to her taste for salt, thus the intake of free-choice mineral and, subsequently, the mineral status of cows within a herd can be variable [6]. Injectable trace minerals can be used to provide additional supplemental trace minerals and allow for a known amount of trace minerals to be delivered to each animal. Trace mineral injections have been proven to increase the status of animals in the short term [7,8].

However, little research has been conducted to evaluate the effects of supplementation of trace minerals through an injection in cow–calf production systems when free-choice mineral is provided. Mundell et al. [9] reported improved conception to fixed-time artificial insemination in beef cows given trace mineral injections 105 days before calving and again 30 days before breeding, when grazing native range in Kansas, USA. However, after exposure to clean-up bulls, no difference in overall pregnancy rate was detected. The calves nursing injected dams also received trace mineral injections at birth and at approximately 71 days of age, but no improvements in their growth were observed. However, in Illinois, USA, Stokes et al. [10] reported a reduced fixed-time artificial insemination conception rate of first-calf heifers, receiving repeated trace mineral injections, with no differences in overall pregnancy rate after exposure to clean-up bulls. The heifers receiving the trace mineral injection had greater milk production, and their calves had greater Cu and Se status at birth, but no differences in calf growth rates were observed.

The objective of this trial was to determine the effects of a trace mineral injection on the trace mineral status of cows and calves and the resulting impacts on the reproductive performance of the cows and the growth of their nursing calves.

## 2. Materials and Methods

All procedures and protocols were approved by the University of Idaho Institutional Animal Care and Use Committee.

Crossbred beef cows (Angus, Hereford, and Angus × Hereford cross) and their calves located at the Nancy M. Cummings Research Extension and Education Center in Carmen, ID, USA, were used in this study. The trial was initiated in December of 2012. During the summer prior to the initiation of the study, approximately half of the 200 cows used in this study grazed native range, while the remainder were maintained on irrigated pasture. Cows were stratified by their summer grazing regimen, age (range from 2 to 13 years with 24% being 2 to 4, 66% being 5 to 10, and 10% being 11 to 13 years old), and expected calving date and assigned to receive trace mineral injections (TMI) of Multimin 90 (Multimin, Fort Collins, CO, USA) containing 15 mg/mL Cu, 10 mg/mL Mn, 5 mg/mL Se, and 60 mg/mL Zn at pre-calving and pre-breeding or to remain as untreated controls (CON). The cow was considered the experimental unit, and cows remained on the same treatment over the two-year trial.

During the winter and early spring periods (December–May), cows were fed alfalfa hay and wheat straw. In May, the cows began grazing on irrigated orchard grass (*Dactylis glomerata*) pastures and continued grazing these pastures until December. During this time, cows were rotationally grazed on pasture that were approximately 6 ha each. The trace mineral concentration of these forages (Table 1) was analyzed by a commercial laboratory (Cumberland Valley Analytical Services, Hagerstown, MD, USA) using the Metals and Other Elements in Plants protocol [11]. Modifications to the protocol included ashing a 0.35 g sample for 1 h at 535 °C and digesting in open crucibles for 20 min in 15% nitric acid on hotplates. Samples were then diluted to 50 mL and analyzed by inductively coupled plasma spectroscopy.

All forages were deficient in Cu, Se, and Zn. Additionally, the pasture contained moderately antagonistic concentrations of molybdenum. Manganese was adequate in the pasture and would have been marginal in the winter alfalfa hay/straw diet. A custom-made free-choice mineral supplement (Table 2) was offered throughout the entirety of the trial with the exception of a 53-day period in year 1 (32–85 days post artificial insemination) when they were provided with a commercial free-choice mineral supplement (Purina Wind and Rain Storm All Season 7.5 Complete) due to the custom mineral being unavailable. Salt was added to the mineral mixes to achieve a targeted intake of 113 g/cow/day of the mineral mix.

### 2.1. Cow Management Timeline

**Figure 1:**
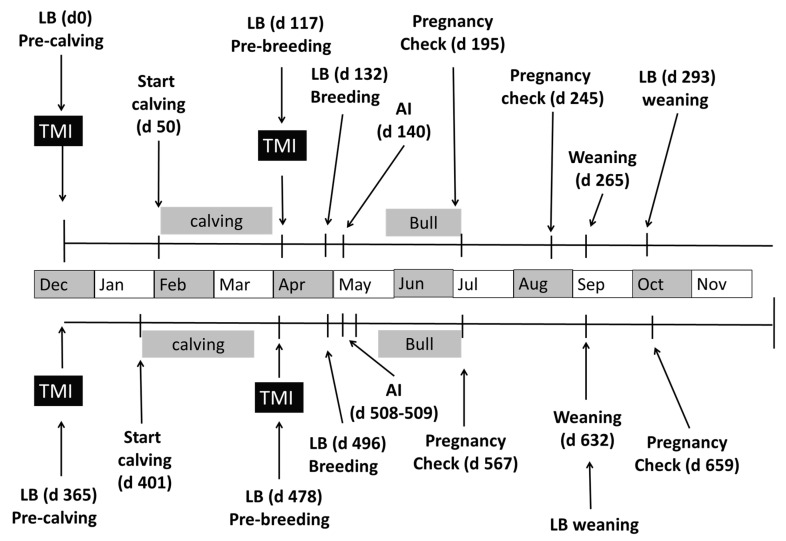
Timeline of two-year trial evaluating the impact of trace mineral injection (TMI) of beef cows on trace mineral concentration in liver (LB) and effects on cow reproduction and calf growth. Injections were given to cows pre-calving (days 0 and 365) and pre-breeding (days 117 and 478), and liver biopsies were taken prior to injection on those dates. Liver samples were also taken 15 to 18 days post pre-breeding TMI (days 132 and 496) and near weaning (days 293 and 632). Cows were estrus synchronized and artificially inseminated (AI), then exposed to fertile bulls. Pregnancy was determined using ultrasonography (days 195 and 567) to determine pregnancy due to AI and by rectal palpation (days 245 and 659) to determine the overall pregnancy rate.

An overview of events during the trial is shown in Figure 1. In December of 2012 (50 days prior to the start of calving season), cows in the TMI group were injected with 6 mL of Multimin 90 (0.67 mL/68 kg of body weight). Then, at 23 days prior to AI (117 d post pre-calving injection), cows in the TMI group were again given 6 mL of Multimin 90 (0.69 mL/68 kg of BW). All cows were estrus synchronized using a 5-day CO-Synch plus CIDR protocol, in which cows were given a 2 mL injection of gonadotropin releasing hormone (GnRH, 43 μg/mL; Fertagyl, Merck Animal Health) and received an insert of a controlled internal drug release device (CIDR vaginal insert containing 1.38 g of progesterone; Eazi-Breed CIDR, Zoetis Animal Health, New York, NY, USA) 8 days prior to AI (day 132). The CIDR was removed 5 days later (day 137) and an injection of prostaglandin F_2α_ (PG; 25 mg; Lutalyse, Zoetis Animal Health) followed by a second PG injection 5.6 h later was administered. Cows were administered an injection of GnRH and inseminated either 72 or 80 h after CIDR removal (day 140) with sexed semen. Cows were stratified across injection treatment to one of the two insemination times (72 or 80 h). Cows were randomly inseminated by one of two technicians and then exposed to fertile bulls for natural-service breeding 17 days after AI (day 158) and remained with the bulls for 45 days. Pregnancy was determined using ultrasonography at 55 days post AI (day 195) and by rectal palpation at 105 days post AI (day 245). The artificial insemination pregnancy rate was calculated using ((n pregnant to AI/total *n* synchronized) × 100).

Some cows (*n* = 14 CON and *n* = 12 TMI) were removed from the study during year 1 due to being culled from the herd for loss of calf (*n* = 1 CON and *n* = 2 TMI) or other management reasons (behavior or conformation). Additionally, cows were culled at pregnancy check in October for failing to conceive. Bred heifers that had received a TMI (*n* = 11) or not (*n* = 10) prior to breeding as heifers in year 1 (but were not a part of this study in year 1) were added to the trial in year 2 starting at the time of the pre-calving injection (day 365; December 2013) to replace cows removed from the study.

In December of 2013 (36 days prior to the start of calving season; day 365), cows in the TMI group were injected with 7.5 mL of Multimin 90 (0.75 ± 0.014 mL/68 kg of BW). Twenty-eight days prior to AI (day 401), cows in the TMI group were given 6.5 mL of Multimin90 (0.75 ± 0.020 mL/68 kg of BW). Cows were estrus synchronized using a modification of the 5-day CO-Synch plus CIDR protocol. All estrous synchronization products used were the same as in Year 1. Briefly, cows received an injection of GnRH and a CIDR insert on day 500. The CIDR was removed 5 days later (day 505), an injection of PG was administered, and an estrus detection aid (EstroTect, EstroTect, Inc. Denver, CO, USA) was applied. Cows with activated detection aids (50% or greater activation) were then inseminated by fixed-time AI at 72 h with non-sexed (*n* = 72 CON and 65 TMI) or sexed semen (*n* = 22 CON and 19 TMI) by one of three technicians (days 508 through 509). Cows that showed no estrous response to synchronization by 72 h post CIDR removal were inseminated by fixed-time AI at 96 h post CIDR removal. Regardless of estrous response, all cows received GnRH at fixed-time AI. Cows were exposed to fertile bulls on day 518 and co-mingled for 49 days. Pregnancy was determined using ultrasonography at 56 days post AI (day 567) and by rectal palpation at 150 days post AI (day 659). Cows that lost calves between calving and breeding in year 2 were culled from the herd (*n* = 3 CON and *n* = 2 TMI). Additionally, cows that were less than 30 days post-calving at the start of the breeding (*n* = 5 CON and *n* = 7 TMI) were not synchronized and were removed from the pregnancy analysis in year 2. To evaluate the potential effect of TMI on timing of conception, change in calving date in year 2 was calculated by subtracting 365 from the number of days between calving in year 1 and calving in year 2.

All cows were evaluated for body condition score (BCS; 1—emaciated, 9—obese), before the start of calving (December), one month prior to the start of breeding (April), and at summer pregnancy check (July).

### 2.2. Calf Management Timeline

Calves were sired by Angus, Hereford, or Simmental bulls, with calves from TMI cows receiving a 1 mL injection of Multimin 90 between birth and 24 h of age in both years. In year 1, TMI calves also received an injection at branding on day 119 of the trial (1 mL/45 kg BW, 49 ± 1.3 days of age (DOA)). In year 1, jugular blood was collected from calves (*n* = 40, 20 per treatment) at branding to determine plasma trace mineral concentrations. The weight of calves was recorded at birth (days 50–110 and days 401–461) branding (day 119; 49 ± 1.3 DOA, year 1 only), summer pregnancy check (days 195 and 567; 128 ± 1.3 DOA, year 1; 128 ± 1.2 DOA, year 2), and at weaning (days 265 and 632; 197 ± 1.3 DOA, year 1; 193 ± 1.2 DOA, year 2). Two-hundred and five day adjusted weaning weights were calculated using the Beef Improvement Federation guidelines [12].

Calves were removed from analysis if they died (*n* = 5 CON and 6 TMI in year 1; 2 CON, and 3 TMI in year 2) or were born as twins (4 and 3 sets of twins for CON and TMI, respectively in year 1). Thus, 91 CON and 91 TMI calves were used to evaluate the effects of TMI in year 1 and 98 CON and 97 TMI calves were used in year 2.

### 2.3. Liver Sampling

Liver biopsies were collected from 20 cows per treatment that were selected randomly. Cows were sampled at pre-calving (days 0 and d 365), pre-breeding (days 117 and 478), 15 to 18 days post pre-breeding TMI (referred to as breeding; days 132 and 496), and near weaning (days 293 and 632) to determine liver trace mineral concentrations. Biopsies were collected from the same cows at each sampling date. Liver biopsies were also collected from the calves (*n* = 40, 20 CON, 20 TMI) of sampler cows at weaning (days 265 and 632). Liver biopsies were collected using the method of Engle and Spears [13]. Liver biopsy samples were placed in a plastic culture tube, transported on ice to the laboratory, and frozen at −20 °C. Liver samples were then dried in a forced-air oven (60 °C). Samples were then shipped to the Diagnostic Center for Population and Animal Health (Lansing, MI, USA) and analyzed for trace mineral concentration. Once at the laboratory, tissues were dried overnight in a 75 °C oven and then digested overnight in nitric acid. Elemental analysis followed the methods of Wahlen et al. [14] using an Agilent 7500ce Inductively Coupled Plasma–Mass Spectrometer (Agilent Technologies Inc., Santa Clara, CA 95051, USA). Elemental concentrations were calibrated using a four-point liver curve of the analyte–internal standard response ratio. The lowest concentrations points were 0.1 µg/mL for Cu and Zn, 0.5 ng/mL for Mn, and 0.1 ng/mL for Se. Standards were from GFS (GFS Chemicals, Powell, OH 46065, USA). A National Institute of Standards and Technology (NIST, Gaithersburg, MD 20899, USA) bovine liver standard was used as a control.

### 2.4. Statistical Analysis

For all models, the individual animal was the experimental unit and significance was declared at *p* ≤ 0.05. Due to the lower number of samples and, thus, power, tendencies are discussed when 0.05 < *p* ≤ 0.15 for all mineral analysis.

Liver mineral concentrations of Cu, Mn, Se, and Zn were analyzed using the mixed procedure of SAS (PROC MIXED; SAS Inst., Inc., Cary, NC, USA) with the fixed effect of treatment, day, and their interaction included in the models. Day was considered a repeated effect. The covariates of initial pre-treatment liver concentrations (day 0) were used as a covariate in all models.

Cow BCSs were analyzed using a mixed-effects model with treatment and day as fixed effects. The treatment by day interaction was not significant (*p* = 0.81) and was removed from the model. Day was considered a repeated effect. Cow BCS recorded on day 0 was used as a covariate.

Pregnancy rates were analyzed using logistic regression (PROC GENMOD; SAS Inst., Inc., Cary, NC, USA). For year 1, the model used to assess differences in AI pregnancy rate tested the effects of treatment and AI timing treatment (72 vs. 80 h) and their interaction. The interaction was not significant (*p* = 0.54) and was removed from the model. The AI timing was not significant (*p* = 0.37) and was removed from the model. The covariates of AI sire and AI technician were tested. The covariate of AI technician was not significant (*p* = 0.19) and was removed from model. The model used to assess differences in overall pregnancy rate included the fixed effect of treatment.

For year 2, the model used to assess differences in AI pregnancy rate included treatment, semen type (sexed vs. non-sexed), and timing of AI (heat response (72 h) vs. delayed 96 h after CIDR removal) and their interactions. There were no significant interactions (*p* > 0.42) and all interactions were removed from the model. The fixed effects of semen type and timing of AI were not significant (*p* ≥ 0.31) and were removed from the model. The covariates of AI sire and AI technician were tested. The covariate of AI sire was not significant (*p* = 0.47) and was removed from the model.

Calf plasma concentrations of Cu, Mn, Se, and Zn, in year 1, were analyzed using the mixed procedure of SAS, and treatment was considered a fixed effect with calf sex being used as a covariate.

Within each year, calf birth weight, average daily gain (ADG), weaning weight, 205-day adjusted weaning weight, and liver mineral concentrations were analyzed using a mixed-effects model including the fixed effect of treatment. Calf sex and dam age were used as covariates for calf birth weight, ADG, and actual weaning weight. Dam age and calf sex were used as covariates for liver mineral concentrations.

## 3. Results and Discussion

### 3.1. Liver Mineral Status of Cows

**Figure 2:**
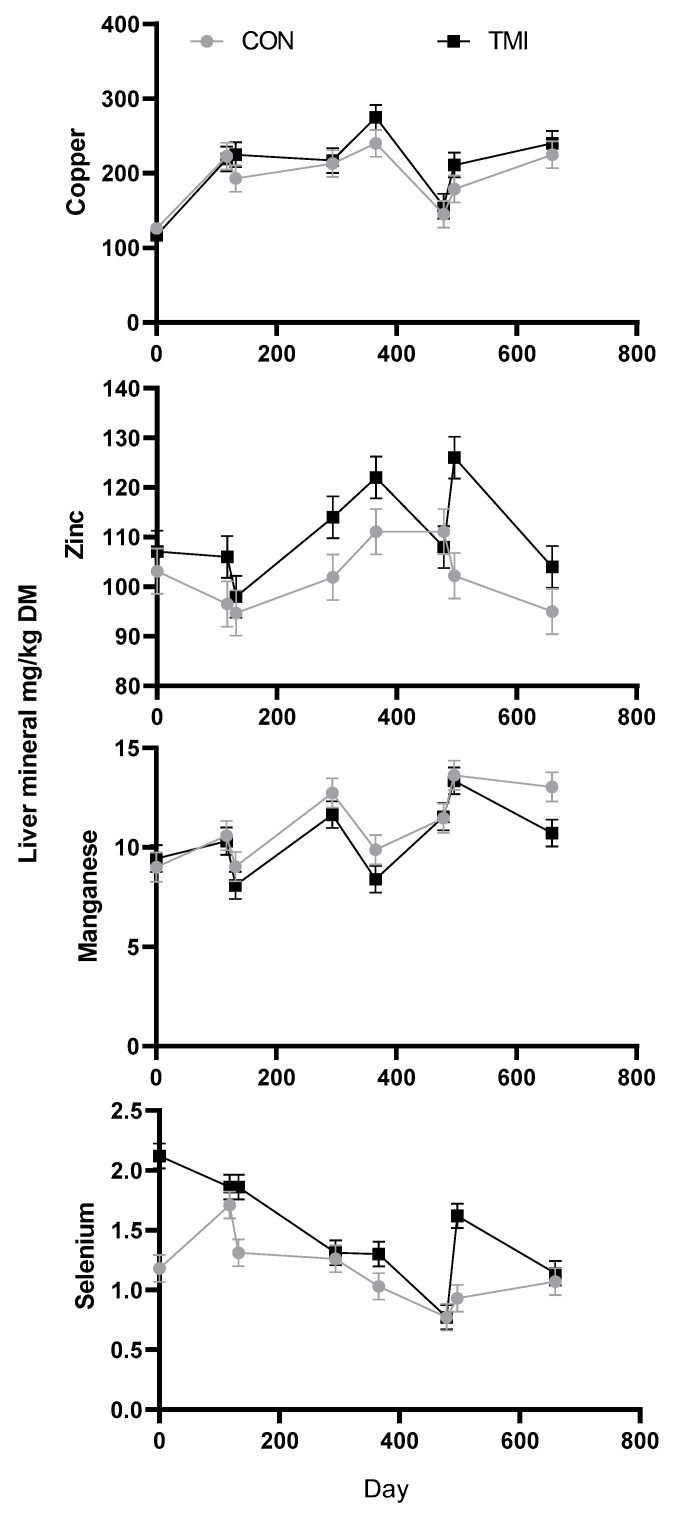
Effect of trace mineral injection (TMI) containing Cu, Se, Mn, and Zn or no injection (CON) on liver concentrations in beef cows over a two-year period. Injections were given pre-calving (days 0 and 365) and pre-breeding (days 117 and 478), and liver biopsies were taken prior to injection on those dates. Samples were also taken 15 to 18 days post pre-breeding TMI (referred to as breeding; days 132 and 496), and near weaning (days 293 and 632). Initial trace mineral concentration (day 0) was used as a covariate. Treatment by day effect not significant (*p* ≥ 0.67) for Cu or Mn but tended to be significant (*p* = 0.13) for Zn and was significant (*p* < 0.01) for Se. Day effect was significant for all minerals (*p* < 0.01). Treatment effect (*p* ≤ 0.02) was significant for Mn, Se, and Zn and tended (*p* = 0.06) to be significant for Cu.

When evaluating the liver mineral concentrations (Figure 2), there was not a significant (*p* ≥ 0.67) treatment by day effect for Cu or Mn. However, treatment by day tended to be significant (*p* = 0.13) for Zn and was significant (*p* < 0.01) for Se. Day effect was significant for all minerals (*p* < 0.01).

There was a tendency (*p* = 0.06) for liver Cu to be increased in TMI vs. CON (221 vs. 203 mg/kg DM, respectively). Increased liver Cu due to TMI has been consistently observed [7,8,10]. However, based on liver Cu concentrations, CON apparently absorbed an adequate amount of Cu from the forage and free-choice mineral, given that their liver Cu was greater than 125 mg Cu/kg DM, which would be considered the lower threshold for adequate status [15].

Liver Zn was increased (*p* < 0.01) by TMI compared to the CON, 111 vs. 102 mg/kg of DM, respectively. However, again both groups would have been considered solidly in the range considered adequate (40–200 mg Zn/kg DM) throughout the trial.

Liver Mn was decreased (*p* = 0.02) by TMI compared to that in the CON with a concentration of 10.6 vs. 11.5, respectively. Others have reported minimal [7] or no [8,16] response in liver Mn to TMI. While it is unexpected for an injection containing Mn to result in reduced liver Mn, it is important to understand that liver Mn often does not reflect dietary intake and, thus, status [15]. In fact, there are currently no well-established criteria for the evaluation of Mn status in beef cattle [17].

Liver concentrations of 1.25 to 0.60 mg Se/kg DM would be considered marginally deficient with less than 0.60 mg Se/kg DM being deficient [15]. The liver Se of the CON was below 1.25 mg/kg DM at the day 132 sampling (breeding in year 1) and remained in the marginal range throughout the study. There was a significant treatment by day effect for Se, which appears to mainly be driven by the fact that the TMI had increased (*p* < 0.01) liver Se at the breeding sampling (days 132 and 478) but not at many of the other time points. The breeding samples were taken 15 and 18 days after the pre-breeding injection. Thus, it appears that the injection resulted in a relatively short-term improvement in Se stores. This may be due to the fact that the Se was being mobilized for use by the animal.

### 3.2. Cow Body Condition

**Figure 3:**
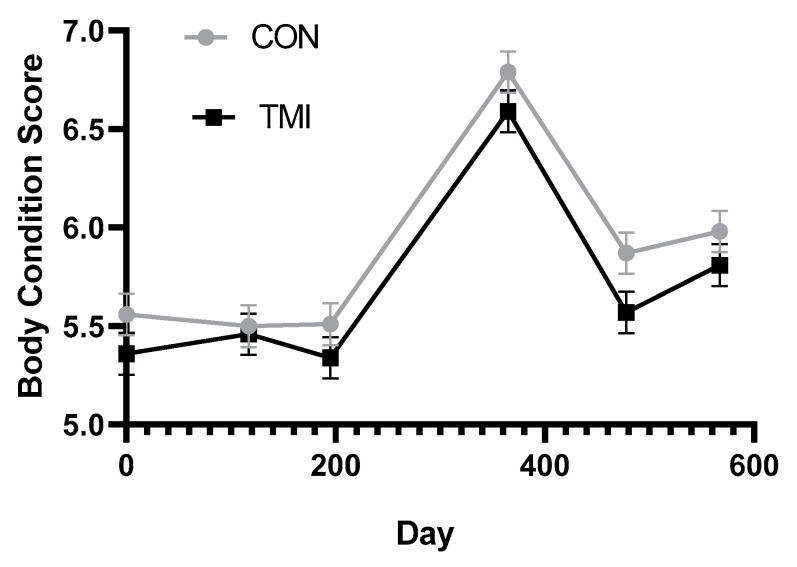
Comparison of trace mineral injection (TMI) or no trace mineral injection (CON) on body condition score (1—emaciated, 9—obese) of cows. Injections were given pre-calving (days 0 and 365) and pre-breeding (days 117 and 478). Day 0 score used as covariate (*p* < 0.01). Treatment by day (*p* = 0.81), treatment (*p* = 0.01), day (*p* < 0.01).

Throughout the trial, cows were in good condition with scores above 5.0 (Figure 3). There was a significant day effect due to cows gaining a full BCS from breeding in July of year 1 (day 195) to pre-calving in December year 1 (day 365) and then losing this extra condition from December of year 1 to pre-breeding in April of year 2 (day 478). The CON cows had greater (*p* = 0.01; SEM ± 0.047) BCS (5.93) than the TMI ones (5.76). Stokes et al., [10] found that repeated TMI of heifers, with good trace mineral status, during development and gestation, resulted in increased milk production. This could explain the slight reduction in BCS for TMI cows in our study.

In contrast to our results, a positive effect of TMI on BCS was observed by Mundell et al. [9]. In their system, cows were grazing native range and had access to free-choice mineral during the pre-breeding period. Although not definitive, pre-treatment serum mineral concentrations (prior to calving in year 1) collected from cows in their study indicated that the CON cows may have been marginal in Cu and Zn. Loss of appetite is a common and early sign of zinc deficiency [18]. Providing Zn though the TMI may have simulated intake and led to the improvement in BCS observed by Mundell et al. [9].

### 3.3. Reproductive Performance

Prior to the pre-calving injection in year 1, cows were blocked by age and expected calving date. Thus, there was no difference (*p* = 0.21) between treatments in the number of days post-partum at breeding in year 1. In year 2, there was no (*p* = 0.48) effect of TMI on calving date (change in calving date for CON = 4 ± 2.6 days and TMI = 6 ± 2.7 days). Therefore, there was no difference (*p* = 0.25) among treatments in the post-partum interval at the time of AI in year 2. Trace mineral injection did not affect the reproductive performance of cows (Table 3) in the current study as both pregnancy due to AI (*p* ≥ 0.58) and overall pregnancy rate did not differ (*p* ≥ 0.36) due to treatment in both years.

Mundell et al. [9] observed that cows on native range receiving TMI had greater fixed-time AI conception rates than those receiving a saline injection (60.2% vs. 51.2%, TMI vs. saline, respectively). However, this improvement did not follow through to overall pregnancy rate in their study. In our study, CON cows were adequate in Cu and Zn. Whereas, in Mundell et al. [9], CON cows could be considered marginal in Cu and Zn. The difference in trace mineral status may explain the differences in responses observed. While there are few studies on the impacts of TMI on pregnancy attainment in beef cows, there have been several in beef heifers. Similar to the current study, no improvement in pregnancy due to AI or overall pregnancy after exposure to bulls had been observed with TMI when heifers already had liver Cu and Zn concentrations that would indicate the diet was providing sufficient amounts [19,20,21]. In contrast, first-calf heifers that had received TMI at three timepoints during development plus an additional three timepoints during gestation had reduced pregnancy due to AI, but again no differences in overall pregnancy rate were observed [10].

### 3.4. Trace Mineral Status of Calves

Plasma Se concentrations were increased (*p* = 0.03) in TMI calves when compared to those in CON calves at branding in year 1 (Table 4). No effect of TMI on plasma Cu (*p* = 0.54) or Mn (*p* = 0.21) was observed at branding in year 1. However, plasma Zn concentrations were increased (*p* = 0.05) in TMI calves when compared to those in CON calves (Table 4). Many studies have investigated the use of a TMI on plasma or serum concentrations in growing cattle. A study investigating TMI use in steers demonstrated an initial increase in plasma Mn, Se, and Zn for up to 10 h post injection due to TMI [7]. At 24 h post injection, plasma Se was still elevated in TMI steers, but Cu, Mn, and Zn were not, and by 8 days post injection, plasma concentrations were similar to those in control steers [7]. In another study, the effect of TMI on plasma concentrations of trace minerals was evaluated over an 85-day period [8]. Concentrations of plasma Mn were increased for up to 8 days in TMI cattle and Se was increased in plasma for up to 15 days in TMI.

Therefore, the increase in plasma Se and Zn in the calves observed 49 days after injection in the current study may have been due to increased transfer in the milk from their dam, which received injections prior to calving and breeding, although this cannot be confirmed as the trace mineral concentrations of milk were not determined in this study. The concentration of Zn in cow’s milk is increased with increasing dietary Zn concentrations [23]. It has also been found that supplementation of Se as selenomethionine will increase Se in milk [24]. Further, it has previously been observed that calves nursing cows supplemented with Se and Cu via a rumen indwelling bolus had greater blood Se concentrations than control calves, indicating that Se was increased in the dam’s milk. Unlike Se, the serum Cu was not increased [25]. Mature milk of beef heifers that had received six TMIs over the course of development and pregnancy with the most recent being 113 days prior to sampling did tend to have increased Zn but not Mn or Se [10].

At weaning, liver Cu tended (*p* ≤ 0.12) to be increased in TMI calves compared to that in CON in both years (Table 5). Liver Mn and Zn were not affected by treatment (*p* ≥ 0.18) in either year. Liver Se was not different (*p* = 0.56) between treatments in year 1 but was increased (*p* = 0.05) by TMI in year 2. These data indicate that repeated injection of dams plus injection of calves at birth with TMI had a long-term effect on the Cu and perhaps Se status of the calves. Calves in both groups had adequate concentrations of liver Cu as they were above 125 mg Cu/kg DM. However, both the TMI and CON calves would be considered marginally deficient in Se at weaning.

### 3.5. Calf Performance

Trace minerals are important for immune responses in cattle. Use of TMI in stressed feeder calves has been shown to decrease morbidity resulting in improved performance [26,27]. However, there was no effect (*p* ≥ 0.24) of TMI on birth BW, actual weaning weight, or average daily gain from birth to weaning of calves in either year (Table 6). There were also no differences (*p* ≥ 0.10) in 205-day adjusted weaning weight. In the current study, the calves would have had minimal immune challenges or stressors, and as a result, incidences of morbidly were low. Similarly, no difference in 205-day adjusted weaning weights between control and TMI calves was observed in the study by Mundell et al. [9]. Their management protocol was similar to the protocol in year 1, as the calves who nursed dams who received a TMI pre- and post-partum, received a TMI at birth and again at 71 ± 21 days of age.

There are multiple challenges with using a free-choice mineral to meet the needs of cows and their calves. One challenge is knowing the amount of each element that needs to be supplemented. While a forage test can help provide some insights, there are still issues with interactions among minerals and antagonisms. It is also impossible to ensure that all cows in the group are consuming the target amount as there appears to be considerable variation in intake of free-choice mineral among animals. However, free-choice mineral supplementation can be a way to meet the mineral needs of the cow herd, when it is formulated and managed correctly. In the current study, cows were grazing in paddocks that were about 6 ha in size and, thus, had to travel limited distances to consume free-choice mineral. Use of an injectable source of Cu, Zn, Mn, and Se under these circumstances appears to be unlikely to result in improvement in cow reproductive or calf growth performance.

## 4. Conclusions

The use of a trace mineral injection containing Cu, Mn, Se, and Zn pre-calving and pre-breeding was effective in enhancing the liver concentration of Cu, Zn, and Se. The use of the trace mineral injection in dams plus the use of an injection at birth enhanced plasma concentrations of Se and Zn at branding and increased liver concentrations of Cu at weaning. However, these data indicate that the trace mineral injections do not improve reproductive performance of cows provided with free-choice mineral resulting in good trace mineral status nor do they improve the pre-weaning performance of their calves.

## Figures and Tables

**Table 1:** Concentrations of trace minerals in orchard-grass-based pasture grazed from May to December and alfalfa hay and wheat straw fed to cows from December to May.

Mineral	Requirement ^1^	Pasture	Alfalfa	Wheat Straw
	mg/kg of DM			
Copper	10	8.7	11	5
Iron	50	130	143	137
Manganese	40	77	29	27
Molybdenum	-- ^2^	2.20	1.57	1.42
Selenium	0.10	0.04	0.04	0.03
Zinc	30	25	22	19

^1^ Based on National Research Council (NRC), 1996. Nutrient requirements of Beef cattle, 7th rev. ed. Natl. Acad. Sci., Washington, DC, USA. ^2^ No requirement for molybdenum.

**Table 2:** Trace mineral concentrations of free-choice supplement ^1^ provided during the study ^2^.

Mineral	Concentration mg/kg	Source
Cu	2000	25% Cu proteinate, 75% Cu sulfate
I	125	Ethylenediamine dihydriodide
Se	38	Sodium selenite
Zn	2000	25% Zn proteinate; 75% Zn sulfate

^1^ Salt was added to the mineral mix to adjust intake to a target of 113 g/cow/d. Vitamins A (222,222 IU/kg), D (22,222 IU/kg), and E (222 IU/kg) were also included in the free-choice supplement from December through May. ^2^ Provided for the entirety of the trial with the exception of a 53-day period (32–85 days post AI) in year 1, during which a free-choice mineral containing 1200 mg Cu/kg from basic copper chloride, 3600 mg Mn/kg from manganese sulfate, 27 mg Se/kg from sodium selenite, and 3600 mg Zn/kg from zinc sulfate was provided.

**Table 3:** Comparison of trace mineral injection (TMI) or not (CON) on reproductive performance of cows.

Item	CON	TMI ^1^	SE ^2^	*p*-Value
	% (No. ^3^)			
Year 1				
AI ^4^ pregnancy	48 (42/86)	44 (39/88)	5.3	0.58
Overall pregnant ^5^	93 (80/86)	93 (82/88)	2.9	0.90
Year 2				
AI ^4^ pregnant	38 (36/94)	39 (33/84)	5.1	0.86
Overall pregnant ^5^	93 (88/94)	90 (76/84)	2.9	0.36

^1^ Cow in TMI treatment received Multimin 90 (~0.72 mL/68 kg/BW), prior to the start of calving season and again prior to the start of breeding. ^2^ SE calculated using Standard Error of Proportion. ^3^ Number of animals observed/number of animals evaluated. ^4^ Artificial insemination (AI). ^5^ Pregnant after AI and exposure to a bull for 45 days (year 1) or 49 days (year 2).

**Table 4:** Effect of trace mineral injection (TMI) ^1^ or no TMI (CON) on calf plasma concentrations ^2^ at branding (49 ± 1.3 days of age) in year 1.

Item	Normal Range ^3^	CON	TMI	SEM	*p*-Value
Copper, mg/L	0.7 to 0.9	0.86	0.90	0.039	0.54
Manganese, ug/L	6.0 to 70.0	12.5	16.4	2.15	0.21
Selenium, ug/L	64 to 140	51.9	61.7	2.66	0.02
Zinc, mg/L	0.8 to 1.4	1.00	1.16	0.056	0.05

^1^ Dams in TMI treatment received 6 mL of Multimin 90, 50 days prior to the start of calving season, and their calves received at 1 mL at birth. ^2^ Calf sex was used as a covariate. ^3^ Adapted from Kincaid [15] and Herdt and Hoff [22].

**Table 5:** Effect of trace mineral injection (TMI) ^1^ on liver trace mineral concentrations of calves at weaning (~195 days of age).

Item	Adequate ^2^	CON	TMI	SEM	*p*-Value
	mg/kg of DM				
**Year 1**					
Copper	>125	141	171	14.5	0.12
Manganese	--	8.8	9.8	0.52	0.21
Selenium	>1.25	0.86	0.92	0.067	0.56
Zinc	>40	109	113	5.00	0.60
**Year 2**					
Copper	>125	160	202	16.8	0.09
Manganese	--	15.6	13.0	1.67	0.29
Selenium	>1.25	0.89	1.04	0.051	0.05
Zinc	>40	107	113	3.31	0.18

^1^ Over a two-year period, dams in TMI treatment received Multimin 90 (~0.72 mL/68 kg/BW), prior to the start of calving season and again prior to the start of breeding. Their calves also received at 1 mL at birth. In year 1, TMI calves also received (1 mL/45 kg BW at 49 days of age). ^2^ Reference ranges as indicated by Kincaid [15].

**Table 6:** Effect of trace mineral injection (TMI) of dams and their calves ^1^ vs. no injection (CON) on calf performance.

Item	CON	TMI	SEM	*p*-Value
**Year 1**				
Steers, % (*n* steers/total calves)	57.5 (50/87)	61.1 (58/95)		
Birth BW, kg	40.3	41.3	0.623	0.24
Day of age at weaning	200	195	1.65	0.06
Actual weaning weight ^2^, kg	271	273	3.05	0.64
Average daily gain ^2^, kg/day	1.16	1.17	0.012	0.32
205-day adjusted weaning weight, kg ^3^	276	283	3.23	0.10
**Year 2**				
Steers, % (*n* steers/total calves)	52.5 (52/99)	52.0 (51/98)		
Birth BW, kg	39.9	40.1	0.595	0.74
Day of age at weaning	195	191	1.94	0.16
Actual weaning weight ^2^, kg	260	257	3.36	0.60
Average daily gain ^2^, kg/day	1.13	1.14	0.013	0.85
205-day adjusted weaning weight, kg ^3^	292	291	3.77	0.54

^1^ Dams in TMI treatment received Multimin 90 (~0.72 mL/68 kg/BW) prior to the start of calving season and again prior to the start of breeding. Their calves also received at 1 mL at birth. In year 1, TMI calves also received (1 mL/45 kg BW at 49 days of age). ^2^ Dam age and calf sex were used as covariates. ^3^ Weaning weights adjusted according to Beef Improvement Federation guidelines to account for dam age, calf age, and calf sex [12].
